# Seroprevalence and Factors Associated with Canine Leishmaniasis in Baringo County, Kenya: Implications for an Integrated One Health Surveillance

**DOI:** 10.3390/vetsci13090857

**Published:** 2026-08-24

**Authors:** Hellen Njeri Maingi, Maingi Ndichu, Richard B. Yapi, James Ng’ang’a Chege, Davis Njuguna Karanja, Bruno Enagnon Lokonon, Cherotich Jesca Tangus, Helena Ngowi, Damaris Matoke-Muhia, Bassirou Bonfoh

**Affiliations:** 1 Department of Veterinary Pathology, Microbiology and Parasitology, Faculty of Veterinary Medicine, University of Nairobi, Nairobi P.O. Box 29053-00625, Kenya; nmaingi@uonbi.ac.ke (M.N.); jchege@uonbi.ac.ke (J.N.C.); dnkaranja@uonbi.ac.ke (D.N.K.); ctangus@uonbi.ac.ke (C.J.T.); 2 Centre d’Entomologie Médicale et Vétérinaire, Université Alassane Ouattara, Bouaké P.O. Box 01 BP 1303, Côte d’Ivoire; richard.yapi@csrs.ci; 3 Laboratoire de Biomathématiques et d’Estimations Forestières, Faculté des Sciences Agronomiques, Université d’Abomey-Calavi, Cotonou P.O. Box 04 BP 1525, Benin; brunolokonon@gmail.com; 4 Department of Veterinary Medicine and Public Health, Sokoine University of Agriculture, Morogoro P.O. Box 3000, Tanzania; helenangowi@gmail.com; 5 Center for Biotechnology Research and Development, Kenya Medical Research Institute, Nairobi P.O. Box 54840-00200, Kenya; dmatoke@kemri.go.ke; 6 Centre Suisse de Recherches Scientifiques en Côte d’Ivoire (CSRS), Abidjan P.O. Box 01 BP 1303, Côte d’Ivoire; bassirou.bonfoh@csrs.ci

**Keywords:** canine leishmaniasis, seropositivity, risk factors, rapid diagnostic test, ELISA, public health risk: one health

## Abstract

Leishmaniasis is an emerging zoonotic disease recognized by WHO as a priority neglected tropical disease (NTD) targeted for elimination by 2030. The disease is transmitted through bites of infected female phlebotomine sand flies and presents in three main forms: cutaneous, mucocutaneous, and visceral leishmaniasis. Visceral leishmaniasis (VL) has two distinct epidemiological cycles: anthroponotic visceral leishmaniasis (AVL) cycle, caused by *L. donovani* and transmitted between humans, and zoonotic visceral leishmaniasis (ZVL) cycle, caused by *L. infantum*, with domestic dogs as the main reservoirs. ZVL represents the most severe clinical form and poses a public health risk due to a high fatality rate. In Kenya, the epidemiology and transmission dynamics of *Leishmania* infection among the canine population in endemic areas such as Baringo are poorly characterized. A cross-sectional epidemiological study was conducted in Baringo County, Kenya, one of the endemic foci for human visceral leishmaniasis. Blood samples were collected from 140 dogs in Rabai, Sabor, Mbechot, Kapkuikui, and Chemolingot and analyzed using Rapid Diagnostic Test (RDT) and Enzyme-linked immunosorbent assay (ELISA). Data on potential epidemiological factors and clinical status for dogs were collected using a questionnaire. Statistical analysis was performed using Pearson’s chi-square (χ^2^) test or Fisher’s exact test to assess associations between *Leishmania* seropositivity and potential risk factors. A stepwise logistic regression procedure with an entry significance level of *p* < 0.20 was employed to identify factors associated with leishmaniasis seroprevalence. Cohen’s kappa (κ) agreement was used to assess the diagnostic performance and accuracy of the two serological tests in the absence of a gold standard. The overall seropositivity in dogs was 7.79% and 12.14% at 95% CI for the RDT and ELISA, respectively. Multivariate analysis identified breed and clinical signs as significant factors associated with *Leishmania* seropositivity in dogs. Local-breed dogs were more likely to be Leishmania seropositive than mixed-breed dogs (OR = 11.92; 95% CI: 1.79–79.37; *p* = 0.010). Dogs without clinical signs exhibited significantly lower odds of ELISA seropositivity (OR = 0.06; 95% CI: 0.01–0.29; *p* < 0.001). Dogs with proper care and housing conditions had reduced risk of *Leishmania* seropositivity compared with poorly managed dogs. Significant association with seropositivity was observed for sex, age, origin, and reason for keeping dogs. Diagnostic evaluation showed that the RDT had an excellent specificity and Positive Predictive Value (100%), with good overall agreement with ELISA, although sensitivity was 64.7%. Hence, RDT can be a reliable alternative screening tool compared with ELISA as the reference standard. The study demonstrated the presence of *Leishmania* infection in the dog population in Baringo County, Kenya, a significant public health risk. Early detection and diagnosis of infections in dogs through an integrated One Health approach during routine surveillance are essential. This will facilitate treatment and/or culling of infected dogs and vector control, subsequently reducing the risk of human transmission. Hence, it will go a long way to achieving WHO’s roadmap of eliminating leishmaniasis by 2030.

## Simple Summary

Leishmaniasis is a neglected tropical disease prioritized by the World Health Organization and transmitted by infected sand flies. Dogs are the primary reservoirs of *Leishmania infantum*, the parasite responsible for zoonotic visceral leishmaniasis, a severe form of the disease. This study assessed the prevalence of canine leishmaniasis in Baringo County, Kenya. Blood samples from 140 dogs were analyzed using rapid diagnostic tests (RDT) and enzyme-linked immunosorbent assay (ELISA). The prevalence of infection was 7.8% by RDT and 12.1% by ELISA. Local dog breeds and those exhibiting clinical signs demonstrated higher infection rates, while adequate care and housing were associated with reduced risk. The RDT demonstrated high specificity and strong concordance with ELISA. These findings confirm the presence of *Leishmania* infection in dogs in Baringo County, indicating a potential public health risk. Enhanced surveillance and control measures, implemented through a One Health approach, are necessary to reduce transmission and advance the World Health Organization’s objective of eliminating leishmaniasis by 2030.

## 1. Introduction

Leishmaniasis is a neglected tropical disease (NTD) caused by protozoan parasites of the genus *Leishmania*. It is transmitted by the bites of infected female phlebotomine sand flies, specifically *Phlebotomus* species in the Old World and *Lutzomyia* species in the New World [1,2]. Transmission involves complex zoonotic and anthroponotic cycles with mammalian hosts and sand fly vectors [3,4,5]. Leishmaniasis continues to pose a significant global public health challenge in Mediterranean, tropical, and subtropical regions.

Clinical manifestations of leishmaniasis include cutaneous, mucocutaneous, and visceral forms. Visceral leishmaniasis (VL), also known as Kala-azar, represents the most severe form and may progress to post-kala-azar dermal leishmaniasis (PKDL). Kala-azar is characterized by systemic dissemination of the parasite to vital organs such as the spleen, liver, and bone marrow, and can be fatal if left untreated [3,6].

The epidemiology of visceral leishmaniasis (VL) is largely determined by the reservoir host involved in transmission. Anthroponotic visceral leishmaniasis (AVL), primarily caused by *L. donovani*, has humans as the primary reservoir. In contrast, zoonotic visceral leishmaniasis is associated with domestic and wild canids, which serve as the principal reservoirs for *L. infantum* infection [7,8]. Domestic dogs (*Canis familiaris*) are recognized globally as the primary reservoirs due to their close association with humans and frequent exposure to infected sand flies. Stray and free-roaming dogs play a particularly significant role in sustaining transmission cycles because they often lack regular veterinary care and preventive interventions [9,10].

Canine leishmaniasis (CanL) is a chronic systemic disease characterized by a spectrum of clinical manifestations, ranging from asymptomatic infection to severe illness. Common clinical signs include weight loss, lymphadenopathy, splenomegaly, anemia, epistaxis, alopecia, and various dermatological lesions [5,7,11]. Notably, a substantial proportion of infected dogs may remain asymptomatic while harboring significant parasite loads in the skin, thereby serving as sources of infection for sand fly vectors and facilitating ongoing transmission. This situation poses a significant epidemiological challenge, especially in endemic regions where asymptomatic dogs function as silent reservoirs for human infection [12,13,14]. Although other animals, including cats, horses, and wild canids, may also harbor *Leishmania* parasites, their definitive role in transmission remains uncertain [8].

CanL has been documented in over 50 countries and is increasingly recognized as a significant veterinary and public health issue. Disease transmission is influenced by factors such as vector abundance, environmental conditions, host susceptibility, canine behavior, and preventive measures [15]. Climate change, urbanization, and increased movement of humans and animals have contributed to the spread of leishmaniasis into new regions by altering the distribution of sand fly vectors [5,6,11]. This geographic expansion presents a substantial threat to public health systems, particularly affecting vulnerable groups including children, the elderly, and immunocompromised individuals, who are at the greatest risk for severe disease progression [7,16].

The seroprevalence of canine leishmaniasis varies substantially across endemic regions, with reported rates ranging from 2% to over 30% in Mediterranean countries using different serological tests such as Indirect Fluorescent Antibody Test (IFAT), enzyme-linked immunosorbent assay (ELISA), and Direct Agglutination Test (DAT) [9]. Ecological and climatic conditions influenced this prevalence [17]. Identified risk factors for canine infection include the extent of exposure to sand flies, housing conditions, the dog’s age, and owners’ implementation of preventive measures [6,18,19]. Phlebotomine sand flies include over 800 species worldwide, grouped into five genera: *Phlebotomus* and *Sergentomyia* in the Old World, and *Lutzomyia*, *Brumptomyia*, and *Warileya* in the New World. About 90 species are recognized as vectors of leishmaniasis and are found in tropical, Mediterranean, and subtropical regions. Proven vectors are restricted to species and subspecies within *Phlebotomus* and *Lutzomyia*, with each species transmitting a single parasite that causes a specific disease. The distribution of these vectors depends on factors such as altitude, vegetation, soil type, climate, and host availability [6,8].

Effective control of canine leishmaniasis remains challenging despite interventions such as insecticide-treated collars, vaccination, reservoir management, and dog culling. These difficulties are primarily attributed to vector adaptability, limitations in available treatments, and the persistence of asymptomatic infections. As many infected dogs do not exhibit clinical signs, laboratory-based diagnosis is critical [5]. ELISA demonstrates high sensitivity and is appropriate for large-scale surveillance. In contrast, rapid immunochromatographic tests offer prompt field-based results but exhibit lower sensitivity, particularly in asymptomatic cases. Given that no diagnostic test achieves perfect accuracy, Bayesian Latent Class Analysis is recommended to enhance diagnostic evaluation and reduce bias [20]. However, the present study evaluated the diagnostic performance of the rK39 RDT and ELISA using Cohen’s kappa (κ) agreement chart [21].

In East Africa, particularly in Sudan, Ethiopia, and Kenya, human VL has traditionally been attributed to *L. donovani,* but the epidemiological role of dogs remains an area of ongoing investigation. However, recent evidence indicates that dogs may also serve as significant reservoir hosts in disease transmission [22]. In Kenya, particularly in Baringo County, the prevalence and epidemiological significance of *L. infantum* infection in dogs are poorly characterized. Climatic changes, alterations in vector ecology, and increasing dog populations may further affect transmission dynamics. The present study aims to address key knowledge gaps concerning the role of dogs as reservoir hosts in endemic, resource-limited pastoral communities of Baringo South [23,24].

This study aimed to determine the seroprevalence of CanL, the potential risk factors associated with infection, and to evaluate the potential role of domestic dogs as reservoirs of *Leishmania* infection in Baringo County, Kenya. The findings are expected to provide epidemiological data to inform integrated One Health strategies for the prevention and control of human and canine leishmaniasis.

## 2. Materials and Methods

### 2.1. Description of the Study Area

The study was conducted in Baringo County, Kenya, which was purposively selected because it is a major endemic area for both VL and CL [24]. Baringo is situated approximately 250 km northwest of Nairobi, at an altitude of 1067 m above sea level, and is characterized by a semi-arid climate. The region experiences high annual temperatures averaging 32.8 ± 1.6 °C and receives an average annual rainfall of 512 mm (ranging from 300 to 700 mm), distributed across two distinct rainy seasons. Baringo County covers approximately 11,075 km^2^ with an estimated human population of 555,561 (KNBS census 2009) [25].

The county contains diverse ecological niches that support the breeding and survival of sand fly vectors, particularly *P. martini* and *P. duboscqi.* These habitats include termite mounds, animal burrows, rock crevices, and dense vegetation such as *Acacia* and *Prosopis juliflora* [26]. Traditional dwellings constructed with mud-plastered or stick walls and grass-thatched roofs are also prevalent. Cracks and crevices within these structures provide suitable resting sites for vectors and have been significantly associated with increased leishmaniasis transmission [23,24,26,27].

The population primarily engages in pastoralism, mixed farming, and livestock keeping, activities that frequently involve extended periods outdoors and close contact with animals [24]. Pastoralism has been identified as a significant socioeconomic risk factor for leishmaniasis in the region. Additionally, cultural practices such as sleeping outdoors and limited community awareness of vector-borne transmission further increase vulnerability to infection [23].

**Figure 1:**
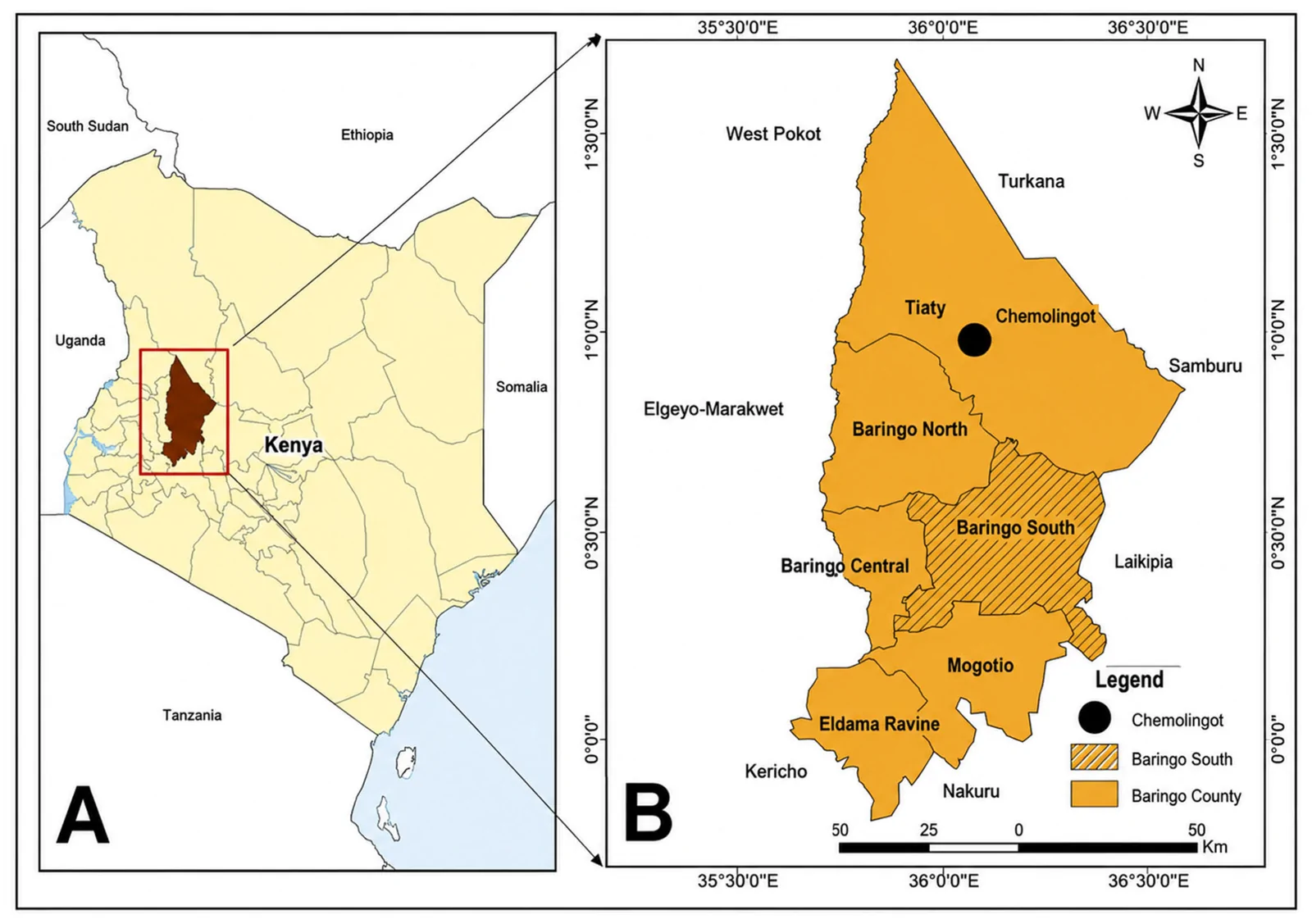
Map of Kenya (**A**) showing the location of Baringo County and inset (**B**) map of Baringo county, showing its sub-counties and the neighboring counties.

Baringo County shares boundaries with the following counties: Turkana and West Pokot to the north, Samburu and Laikipia to the east, Nakuru and Kericho to the south, Uasin Gishu to the southeast, and Elgeyo Marakwet to the west. Administratively, the county is divided into seven sub-counties: Baringo South, Baringo Central, Baringo North, Mogotio, Eldama Ravine, and Tiaty (Figure 1).

### 2.2. Study Design and Selection of Study Sites

A cross-sectional study was conducted in Baringo South and Tiaty Sub-counties to provide a snapshot of the prevalence of visceral leishmaniasis and to assess the distribution of infection in dogs. This methodology is commonly employed in endemic regions to estimate disease prevalence and identify potential reservoirs [28]. The elevated prevalence in the human population within the study area establishes a critical baseline for investigating the role of domestic dogs as reservoirs that maintain transmission cycles in this region [29].

Baringo South and Tiaty sub-counties were purposively selected due to their high documented human leishmaniasis seroprevalence. Site selection further considered logistical feasibility, including accessibility and the need to maintain adequate security considering regional tribal conflicts and cattle rustling, to ensure safe access to affected populations [30]. Within Baringo South sub-county, four locations—Marigat, Kimalel, Sandai, and Kapkuikui—were purposively selected based on their epidemiological significance. Marigat was identified as a high-burden hotspot for leishmaniasis [31]. Kimalel was selected from earlier findings of the established center that acted as a primary regional treatment and research center [30]. Sandai and Kapkuikui were chosen due to their endemic lowland ecology and proximity to villages with high documented human seroprevalence [29], and with earlier evidence of spatially clustered endemic villages in Baringo [28].

In Tiaty sub-county, Chemolingot has been recognized as a well-documented endemic focus of human VL. Epidemiological surveillance and laboratory records from Chemolingot Sub-County Hospital consistently demonstrate a substantial disease burden in this area [29]. Additionally, a study of Chemolingot residents found a high positivity rate among symptomatic individuals, with 39.6% testing positive using rK39 antigen kits.

#### 2.2.1. Sampling Strategy

A multistage random sampling technique was employed to select study participants. Initially, sub-locations within Baringo South were identified and stratified into four geographical zones according to ecological and epidemiological characteristics relevant to *Leishmania* transmission. Villages were then selected through a simple randomization procedure. Human leishmaniasis case records from Kimalel Health Center, Marigat Sub-County Hospital, and Chemolingot Sub-County Hospital guided the choice of specific study sites. One sub-location from each target area—Rabai, Sabor, Mbechot, Kapkuikui, and Chemolingot—was included based on the reported incidence of leishmaniasis.

#### 2.2.2. Household Selection

A sampling frame was constructed using household registers obtained from village elders, local authorities, and the Kenya National Bureau of Statistics. Eligible households were selected through simple random sampling with computer-generated random numbers. Due to challenges in obtaining an accurate list of all dogs, a multi-stage cluster sampling approach was implemented to ensure effective representation of dog samples by randomly selecting villages, followed by households or dogs within those villages [32]. The local administrators and community health workers conducted community sensitization and mobilization activities. Written informed consent was obtained from all participants and dog owners before enrollment.

### 2.3. Inclusion Criteria

Households were eligible for inclusion only if both residents and their dogs had resided in the study area for at least 1 year, thereby ensuring that detected infections reflected local transmission. The study included all available dogs over 6 months old in selected households that were accessible, i.e., owned, roaming, or stray. These dogs underwent clinical examination for signs of canine leishmaniasis, including lymphadenopathy, skin lesions, and weight loss, and had blood samples aseptically collected for serological testing.

For the owners’ dogs, the owners must be willing to allow their dogs to participate in the study through blood sampling. In contrast, dogs with a history of excessive aggression were excluded.

### 2.4. Sample Size Determination

The sample size (N) for canine sampling was determined using the formula for estimating prevalence in a cross-sectional study described by [33], based on seroprevalence of 21.3% from a similar study in Egypt [34].

$$n=\frac{Z^{2}pq}{e^{2}}$$

A 7% margin of error was selected instead of the standard 5% to accommodate logistical challenges, low population density, and the mobile nature of pastoralist populations in Baringo South sub-county [29,35].

Where

(*n*) = required sample size

(*Z*) = 1.96, standard normal deviate at 95% confidence interval

(*p*) = 0.213 was the baseline prevalence

(*q*) = 1 − *p*

(L) = desired precision (0.07)

Using these parameters, the calculated sample size was 131 dogs, but we collected samples from 140 dogs.

### 2.5. Ethical Considerations

Ethical approval for this study was obtained from the Ethical Committee for Animal Experimentation of the Faculty of Veterinary Medicine, University of Nairobi (REF: FVM BAUEC/2024/582). All animal handling procedures, clinical examination, and biological sample collection were conducted in accordance with relevant institutional guidelines and regulations. Informed consent was also obtained from all study participants, and verbal informed consent was obtained from all dog owners who agreed to voluntarily permit their dogs to participate in the study and to have blood collected.

### 2.6. Data Collection and Variables

A structured questionnaire was also administered to dog owners to collect data on relevant epidemiological risk factors associated with CanL. Data collected included demographic characteristics of dogs, such as geographical location, age, sex, and breed. In the absence of birth records for most dogs, age was estimated using dental formula, ocular, and musculoskeletal indicators. Information on management and environmental factors, including housing conditions (e.g., indoor housing, outdoor confinement, or earthen-floor shelters), veterinary care such as the use of protective measures against vector-borne diseases like insecticides, and the primary reason for keeping the dog (e.g., hunting, livestock herding, companion, or security). Additionally, the dogs were also examined for clinical signs suggestive of CanL, such as lymphadenopathy, dermatological lesions, alopecia, weight loss, and other relevant clinical manifestations, and data of each dog were recorded.

### 2.7. Blood Sample Collection and Processing

Blood samples were collected from restrained dogs using aseptic techniques and appropriate personal protective equipment (PPE). Approximately 5 mL of blood was drawn from the cephalic or lateral saphenous vein with sterile Vacutainer needles following disinfection with 70% isopropyl alcohol by a qualified veterinarian. Samples were labeled and transported under refrigerated conditions to the Baringo South Veterinary Laboratory. Blood samples were allowed to clot at room temperature to ensure complete coagulation before centrifugation, thereby preventing latent fibrin formation in the serum. Following clotting, samples were centrifuged for 10 min at 2000–3000 rpm to obtain serum for rapid tests and ELISA. Serum aliquots were transferred into cryovials and stored at −20 °C. Subsequently, serum samples were transported to the Kenya Medical Research Institute (KEMRI) for serological testing. All procedures for handling, storage, and transportation adhered to established quality assurance and biosafety protocols.

#### 2.7.1. Rapid Diagnostic Testing (rK39 RDT)

Canine serum samples were initially screened for the presence of anti-*Leishmania* antibodies using the rapid diagnostic test (RDT) for IgG or IgM, following the manufacturer’s instructions for Speed Leish K^®^. The rK39 immunochromatographic assay is commonly used for the serodiagnosis of VL due to its simplicity, rapid turnaround time, and high diagnostic accuracy. The test used in this study demonstrated a reported diagnostic sensitivity of 82.4% and specificity of 99%.

In brief, the required volume of serum was applied to the sample well cassette, followed by the addition of assay buffer. Results were interpreted within the manufacturer-specified timeframe. Samples displaying both control and test lines were classified as positive for anti-*Leishmania* antibodies, while those showing only the control line were classified as negative. Tests without a visible control line were deemed invalid and repeated. All samples that tested positive on the rK39 RDT were aliquoted and stored at −80 °C for subsequent confirmatory ELISA analysis.

#### 2.7.2. Serological Analysis Using Indirect ELISA

Anti-*Leishmania* antibodies in canine serum samples were detected using the VetLine *Leishmania*, Ref. LEIVT0310, Gold Standard Diagnostics, Germany, following the manufacturer’s instructions. This indirect enzyme-linked immunosorbent assay enables qualitative detection of antibodies against *Leishmania* spp. in canine serum samples. The samples, along with positive, negative, and cut-off controls supplied with the kit, were added to a 96-well microtiter plate pre-coated with *Leishmania* antigen. The plates were incubated under manufacturer-specified conditions to facilitate binding of *Leishmania*-specific antibodies in the samples to the immobilized antigens.

After incubation, wells were washed to remove unbound material, and enzyme-conjugated anti-canine immunoglobulin was added. Following a second incubation and wash, tetramethylbenzidine (TMB) substrate solution was introduced, producing a colorimetric reaction proportional to the amount of antibody bound. The reaction was terminated with the stop solution provided in the kit, and optical density (OD) values were measured at 450 nm using a microplate ELISA reader, as recommended by the manufacturer.

Results were interpreted according to the manufacturer’s criteria, utilizing the calculated cut-off value and control performance parameters. Samples with OD values exceeding the established cut-off were classified as positive, while those below the threshold were classified as negative. Quality control was confirmed by verifying that all positive, negative, and cut-off controls met the manufacturer’s acceptance criteria before interpreting sample results.

The VetLine *Leishmania* ELISA demonstrates a reported diagnostic sensitivity of 95.8% (95% CI: 90.47–98.62%) and a diagnostic specificity of 95.43% (95% CI: 91.19–98.01%) for *anti-Leishmania* antibodies in canine samples. Positive control sera were sourced from dogs previously confirmed to be infected with *Leishmania* spp. through parasitological, serological, molecular, and clinical diagnostic methods. Sera from dogs originating from non-endemic areas served as negative controls.

### 2.8. Data Management and Statistical Analysis

Data were entered, cleaned, coded, and validated before analysis. Statistical analyses were performed using Stata version 14 (Stata Corp LLC, College Station, TX, USA). Data completeness was assessed through descriptive checks and missing-value analysis. Descriptive statistics were used to summarize the characteristics of the study population. Continuous variables, including age, were summarized using measures of central tendency and dispersion, while categorical variables were presented as frequencies and percentages [36]. Dog age was subsequently categorized into three groups: young (≤1 year), mature (>1 to <5 years), and old (≥5 years) [9].

The prevalence of *Leishmania* infection was estimated separately for the rK39 rapid diagnostic test (RDT) and ELISA by calculating the proportion of positive dogs among all tested animals [37]. Corresponding 95% confidence intervals (95% CI) were computed [38]. Associations between *Leishmania* seropositivity and potential factors, including sex, age category, housing system, purpose of keeping dogs, breed, clinical status, veterinary care such as ecto-parasite use, and geographical origin, were initially assessed using contingency table analyses [37]. Pearson’s chi-square test or Fisher’s exact test, where appropriate, was used to evaluate differences in prevalence between categories [39].

A stepwise logistic regression procedure with an entry significance level of *p* < 0.20 was employed to identify the most parsimonious model and determine independent predictors of ELISA positivity [40]. Model performance was evaluated using the likelihood ratio test, and goodness of fit was assessed using the Hosmer-Lemeshow test [41]. The discriminatory ability of the final model was evaluated through receiver operating characteristic (ROC) curve analysis, and classification accuracy was assessed using classification tables [42]. Agreement between the rK39 RDT and ELISA results was evaluated using Cohen’s kappa (κ) statistic, complemented by cross-tabulation analyses [21,43]. All statistical tests were two-sided, and statistical significance was defined at *p* < 0.05.

## 3. Results

### 3.1. Potential Risk Factors for Leishmania Seropositivity

The descriptive characteristics of the study population, including origin, age, sex, housing, reasons for keeping dogs, clinical status, veterinary care, insecticide use, and breed, are summarized in Table 1. A total of 140 dogs were enrolled and sampled during the study. Most of the dogs originated from Sabor (45; 32.14%), followed by Mbechot (40; 28.57%), Rabai (27; 9.29%), Chemolingot (16; 11.43%), and Kapkuikui (12; 8.57%).

### 3.2. Prevalence and Factors Associated with Leishmania Seropositivity

The serological tests confirmed active exposure of dogs to *Leishmania* infection in Baringo County. The results from both the rK39 RDT and ELISA tests demonstrated ongoing exposure of dogs to *Leishmania* spp. in Baringo County. In fact, the overall seropositivity of CanL, determined using the rK39 RDT, was 7.86% [95% CI: 4.38–13.70], whereas the estimated seroprevalence using the ELISA was 12.14% [95% CI: 7.65–18.73].

Bivariate analysis using Fisher’s exact test with ELISA test results identified several factors associated with *Leishmania* seropositivity among dogs in Baringo County (Table 2). Among the variables examined, geographical origin, clinical status, breed, and housing conditions were significantly associated with seropositivity. In contrast, sex, age, vaccination status, insecticide use, and the primary reason for keeping dogs were not. Although a greater proportion of seropositive dogs were males (82.4%) than females (17.6%), the association between sex and seropositivity was not statistically significant (*p* = 0.560).

Similarly, seropositivity tended to increase with age, with older dogs accounting for 41.2% of positive cases, followed by mature (35.3%) and young dogs (23.5%); however, these differences were not statistically significant (*p* = 0.760). A significant association was observed between geographical origin and seropositivity (*p* = 0.005). The highest proportion of seropositive dogs was observed in Mbechot (41.2%), followed by Rabai (29.4%) and Kapkuikui (23.5%), whereas only one positive dog was detected in Sabor (5.9%) and none in Chemolingot. Many infected dogs appeared clinically healthy, while others exhibited clinical signs such as emaciation, alopecia, and diarrhea. Although these clinical manifestations may result from conditions other than leishmaniasis, they were significantly associated with *Leishmania* seropositivity (*p* < 0.001). Dogs presenting with emaciation accounted for nearly half (47.0%) of all seropositive animals, while alopecia and diarrhea were observed in 11.8% and 5.9% of positive dogs, respectively. In contrast, 35.3% of seropositive dogs exhibited no apparent clinical signs, indicating asymptomatic infections in the canine population. Breed was also significantly associated with seropositivity (*p* = 0.010). Local breeds accounted for 76.5% of seropositive dogs, compared with 23.5% among mixed-breed dogs. Housing conditions significantly influenced seropositivity (*p* = 0.042). Most seropositive dogs were free roaming (88.2%), whereas only 5.9% were housed in kennels and 5.9% were kept in household compounds. No seropositive dogs were detected among those housed in animal sheds. The higher infection frequency among roaming dogs may reflect increased exposure to sand fly vectors and contact with infected reservoir hosts. Although most seropositive dogs lacked veterinary care, such as insecticide treatment (94.1%), neither dogs with proper veterinary care nor those without were significantly associated with seropositivity (*p* = 1.00 for both variables).

Likewise, dogs kept for hunting (58.8%) and grazing (29.4%) appeared to have higher seropositivity than those kept as pets or for security purposes (5.9% each), but these differences were not statistically significant (*p* = 0.397). Overall, geographical origin, clinical status, breed, and housing system emerged as important factors associated with *Leishmania* seropositivity at the bivariate level and were therefore considered candidates for inclusion in subsequent multivariable analyses (Table 2).

### 3.3. Multivariable Logistic Regression for the Potential Factors Associated with Leishmania Seropositivity in Dogs

A stepwise multivariable logistic regression model was fitted to identify independent predictors of *Leishmania* seropositivity among dogs. The final model included clinical status, geographical origin, housing conditions, breed, and vaccination status. Overall, the model was statistically significant (Likelihood Ratio χ^2^ = 28.12, df = 5, *p* < 0.001), indicating that the included variables collectively explained variation in ELISA seropositivity. The model yielded a pseudo-R^2^ of 0.294, indicating a moderate fit and that approximately 29.4% of the variation in seropositivity was explained by the variables included.

The final multivariable logistic regression identified breed and clinical status as significant predictors of ELISA positivity among dogs. Clinical status emerged as an independent predictor of *Leishmania* seropositivity. Dogs without clinical signs suggestive of canine leishmaniasis had significantly lower odds of ELISA seropositivity compared with dogs presenting clinical manifestations (OR = 0.06, 95% CI: 0.014–0.289, *p* < 0.001). Specifically, asymptomatic dogs had approximately 94% lower odds of infection than clinically affected dogs.

Breed was also significantly associated with infection status. Local dog breeds had substantially higher odds of seropositivity than mixed breeds (OR = 11.92, 95% CI: 1.79–79.37, *p* = 0.010). This finding indicated that local dog breeds were nearly 12 times more likely to be ELISA-positive than mixed breeds. However, the relatively wide confidence interval suggests limited precision of the estimate, likely due to the small number of seropositive animals within some breed categories.

Geographical origin showed a tendency to be associated with *Leishmania* seropositivity. Dogs originating from Rabai had approximately 4.6-fold higher odds of seropositivity compared with those from the reference location (Kapkuikui) (OR = 4.60, 95% CI: 0.78–27.15, *p* = 0.092). Although suggestive of increased risk, the association did not reach statistical significance because the confidence interval included unity. This finding is considered hypothesis-generating and requires validation in larger studies.

Similarly, dogs confined within the household compounds had lower odds of seropositivity than free-roaming dogs (OR = 0.13, 95% CI: 0.01–1.51, *p* = 0.103), indicating a possible protective effect of confinement. Nevertheless, this association was not statistically significant after adjustment for other covariates.

Veterinary care was not significantly associated with *Leishmania* seropositivity. Dogs with proper veterinary care showed lower odds of infection than those without (OR = 0.12, 95% CI: 0.005–2.69, *p* = 0.181). Still, the wide confidence interval and non-significant *p*-value indicate insufficient evidence to support a protective effect in this study population (Table 3).

### 3.4. Area Under the Curve After Logistic Model with ELISA Positive

The logistic regression model demonstrated excellent discriminatory performance, with an area under the ROC curve (AUC) of 0.85, indicating good ability to distinguish ELISA-positive from ELISA-negative dogs.

### 3.5. Agreement Between RDT Positive and ELISA Positive

There was strong concordance observed between the two diagnostic methods (κ = 0.763, *p* < 0.001. The overall observed agreement between the tests was 95.7%.

Cross-tabulation of the results revealed that 123 dogs were negative by both RDT and ELISA, while 11 dogs were positive by both methods. Six dogs tested negative by RDT but positive by ELISA, whereas no dogs were positive by RDT and negative by ELISA. These findings indicated that the RDT produced no false-positive results but failed to identify a proportion of ELISA-positive animals. Using ELISA as the reference test, the RDT demonstrated a sensitivity of 64.7% and a specificity of 100%. The positive predictive value was 100%, showing that all dogs testing positive by RDT were confirmed positive by ELISA. The negative predictive value was 95.3%, indicating that most RDT-negative dogs were truly negative. However, the occurrence of six false-negative results suggests that the RDT failed to detect approximately one-third of ELISA-positive dogs.

Overall, the rK39 RDT exhibited excellent specificity and positive predictive value, together with substantial agreement with ELISA. These findings suggest that a positive RDT result is highly reliable for identifying *Leishmania*-seropositive dogs. However, because of its moderate sensitivity, a negative RDT result should be interpreted cautiously, and confirmatory testing using more sensitive serological assays such as ELISA may be warranted in epidemiological surveys and surveillance programs.

## 4. Discussion

This study is the first epidemiological assessment of factors associated with *Leishmania* seropositivity in dogs in Baringo County, Kenya. These findings highlight that seropositive dogs are clinically and epidemiologically important in endemic areas like Baringo by maintaining environmental infection, irrespective of the presence of clinical disease [15,16]. However, seropositivity only demonstrates previous exposure to *Leishmania* infection. Therefore, confirming the *Leishmania* species through isolation and PCR characterization is essential to assess the potential role of these dogs as a principal reservoir host [44].

The study identified an overall seropositivity of 7.86% with the Rapid Diagnostic Test and 12.14% with ELISA. The observed seroprevalence is consistent with findings from other endemic regions where similar serological diagnostic methods were used to detect specific antibodies produced by the canine immune system in response to *Leishmania* antigens [12,17]. These endemic areas include Zambia [16], Portugal using DAT [17], Bangladesh with DAT [44], Turkey via the IFAT [45], Sardinia, Italy, using IFAT [46], Palestine using ELISA [47], northeastern Portugal by ELISA and DAT [48], France by in-house ELISA [12]. Higher seropositivity has been reported in Northern Egypt using ELISA [34], China using ELISA [49], and Ethiopia using ELISA, where dogs over 6 months of age, who were accessible and whose owners were willing to be sampled, were recruited in the study, while dogs that were sick and under treatment were omitted [9]. These differences in seropositivity across geographical regions are primarily attributed to ecological factors, vector abundance, and climatic conditions that affect vector breeding and dog exposure [46].

Marked spatial variation in seropositivity was observed across the five study sites, with the highest seropositivity reported in Mbechot (41.2%), Rabai (29.4%), and Kapkuikui (23.5%), whereas no seropositive dogs were detected in Chemolingot. Similarly, dogs from Rabai had approximately 4.6 times higher odds of ELISA positivity than those from Kapkuikui; however, this was not associated with infection. The observed spatial heterogeneity is consistent with prior evidence demonstrating significant micro-geographic heterogeneity in *Leishmania* transmission within Baringo [27,28]. Comparable geographical clustering has been reported in Ethiopia [9], Zambia [16], Portugal [17], and Palestine [47]. Such micro-spatial heterogeneity likely reflects local differences in rural or peri-domestic conditions, such as vegetation and livestock management, that influence vector density and vector–host contact [17].

The principal finding of this study was the association between dogs’ clinical status and *Leishmania* seropositivity. Dogs presenting with compatible clinical signs were significantly more likely to be ELISA seropositive, while dogs without clinical signs exhibited significantly lower odds of ELISA positivity (OR = 0.06). The observed correlation aligns with findings from studies conducted in Brazil, in which symptomatic dogs had a significantly higher risk of testing positive (OR = 1.8) than asymptomatic dogs [50]. In canine leishmaniasis, elevated antibody levels are detected in dogs presenting with weight loss, alopecia, and lymphadenopathy, while in chronic infections antibody levels may remain elevated, supporting their utility as biomarkers for monitoring disease progression [51]. A similar association has been reported in Portugal [48], Iran [52], Tunisia [52], and Italy [46]. These results indicate that clinical manifestations are indicative of advanced infection and enhance the likelihood of serological detection [4]. Nevertheless, a substantial proportion of seropositive dogs were clinically asymptomatic, supporting prior evidence that asymptomatic infections are common in endemic regions, indicating the presence of hidden or subclinical infections. These findings are comparable with those reported in Palestine [47], Brazil [53], and Zambia [16]. Asymptomatic dogs are epidemiologically important because they can infect sand flies and sustain silent *Leishmania* transmission despite the absence of clinical signs [16]. These subclinical infections may subsequently progress to overt disease following immunosuppression or concurrent infections [13]. Consequently, surveillance based solely on clinical examinations is likely to underestimate the true burden of infection and overlook important reservoir hosts.

Breed was another significant predictor of *Leishmania* seropositivity, with local dog breeds exhibiting nearly 12 times higher odds of ELISA seropositivity than mixed breeds. This association is most likely explained by management practices rather than inherent genetic susceptibility, as local breeds are commonly used for herding and hunting and spend prolonged periods outdoors, thereby increasing exposure to infected sand flies. A similar association has been reported in Turkey [45] and Italy [46]. Conversely, research from Egypt identified greater susceptibility among pure breeds, including German Shepherds and Rottweilers [34] and Alabama [18], indicating that host genetics and environmental exposures may influence breed-related susceptibility. In addition, poor management practices and lack of veterinary care, particularly the use of insecticides, repellents, and vaccinations, have a greater impact than breed alone. Studies from Portugal demonstrate that dogs receiving regular veterinary care, such as those in managed shelters employing preventive measures with anti-feeding effects, often exhibit lower seroprevalence than domestic dogs with less oversight [48].

Male dogs in this study exhibited higher seropositivity than females. This finding aligns with previous reports from Egypt [34], Italy [46], and Brazil [19]. The observed pattern may be attributed to behavioral differences, particularly increased roaming among males driven by territorial and reproductive behaviors, which could increase exposure to infected vectors. In addition, older dogs demonstrated higher seropositivity than younger dogs. Although older and male dogs showed higher seropositivity, neither variable remained independently associated with infection after adjustment. Our results do align with a study in Bingöl, Turkey [45] but contrast with findings in Egypt [34] and Sardinia, Italy [46] where older dogs and males often show significantly higher likelihoods of infection (up to 12 times higher in some age groups). Similar inconsistencies have been reported across endemic settings in Portugal [48], Iran [52], Egypt [34], Palestine [47], and Italy [46]. The higher seropositivity observed in older dogs is likely attributable to cumulative exposure to infected sand flies, resulting in increased seroconversion and persistent antibody responses [53,54,55]. However, contrasting findings from Turkey, where younger dogs exhibited higher seropositivity [45], suggest that age-related seropositivity patterns vary across epidemiological settings. Nevertheless, dogs of all age groups remain susceptible to *Leishmania* infection in endemic areas.

Dogs used for outdoor activities such as hunting and herding animals were at higher risk of *Leishmania* seropositivity than those that spend more time indoors primarily for security or companionship. This occupational risk is consistent with findings from Portugal [48], Sardinia, Italy [46], Ethiopia [9] and Alabama [18]. The elevated risk among these working dogs engaged in outdoor activities is due to significantly greater exposure to infected sand flies, stemming from their prolonged presence in sylvatic or peridomestic environments, where vector activity is most intense.

Dogs that received little or no veterinary care exhibited higher seropositivity than those that did. Comparable findings have been reported in Studies conducted in Egypt [34], Portugal [48], and Brazil [56]. Vaccination has also been shown to significantly reduce the risk of progression to clinical disease (OR = 3.8) in naïve dogs exposed during transmission seasons [57]. Collectively, these findings indicate that although biological factors such as age and sex may vary regionally, consistent veterinary management is a universal deterrent to the spread of CanL [17]. This indicates that regular ectoparasite control, insecticide-treated collars, vaccination, and routine veterinary management substantially reduce the risk of *Leishmania* seropositivity. These findings emphasize that preventive veterinary care represents an important and practical intervention for reducing CanL and interrupting zoonotic transmission.

Confinement within the household compound was associated with lower odds of *Leishmania* seropositivity than free-ranging status. However, the association was not statistically significant. This finding is consistent with previous canine risk-factor analyses, which indicated that dogs housed in enclosed courtyards have reduced exposure to sand flies compared to free-roaming dogs [27]. These results further provide evidence that housing and management practices influence the peridomestic transmission cycle [56]. This trend is consistent with findings from Ethiopia, where a mixed indoor-and-outdoor living environment was an independent predictor of infection (OR = 2.19), and outdoor housing was associated with increased exposure [9]. Furthermore, roaming dogs may experience increased exposure to sandfly vectors due to sedentary outdoor lifestyles, rural environments, and inconsistent preventive treatment [48].

Diagnostic evaluation demonstrated important differences between the two serological assays. The RDT demonstrated excellent specificity (100%) but only moderate sensitivity (64.7%) in this study. All RDT-positive dogs were confirmed positive by ELISA, resulting in a 100% positive predictive value. However, the RDT failed to detect approximately 35% of animals identified as seropositive by ELISA. This diagnostic discrepancy highlights a significant limitation: although a positive RDT result is highly reliable for identifying infected dogs in the field, a negative result does not definitively exclude infection [56]. Similar findings have been reported in other endemic regions, including Zambia [16] and Italy [46], where differences in sensitivity among diagnostic methods, as in the study comparing ELISA vs PCR-RFLP, lead to undetected infections in seronegative animals. Therefore, while serological tests such as RDT and ELISA are useful for rapid field assessment in our study, their limited sensitivity reduces their suitability as standalone tools for comprehensive screening or surveillance programs targeting elimination. Similar findings have been reported in Brazil, where various serological tests, such as IFAT, DAT, sELISA, and RDT, demonstrated varying sensitivity and specificity [58]. Seronegative dogs by RDT may still harbor infection, indicating hidden infection; hence, limitation of antibody-based screening alone [44,49].

In contrast, ELISA demonstrated superior diagnostic sensitivity by detecting significantly more infected dogs than the rapid diagnostic test (RDT) [59]. The data indicated that ELISA could detect low antibody levels, which are frequently observed in asymptomatic carriers. These findings are consistent with previous studies conducted in Brazil [58], Turkey [45], Portugal [48]. This evidence reinforces the role of ELISA as a “gold standard” for mass screening in field epidemiological studies, particularly in high-transmission areas such as Turkey [45], Egypt [34] and Brazil [58]. Additionally, because many dogs in endemic regions such as Baringo possess antibodies from prior exposure or subclinical infection rather than from active disease, ELISA offers an effective means to assess the parasite’s true prevalence in the population accurately [58]. ELISA detected substantially more infected animals, particularly those with low antibody titers associated with asymptomatic infection, making it better suited for epidemiological surveillance. Nevertheless, neither assay can distinguish active infection from prior exposure, and currently there is no universally accepted gold standard for the diagnosis of canine *Leishmania* infection because no single diagnostic test can accurately detect the full spectrum of infection, from early exposure and asymptomatic carriage to overt clinical disease [20].

The logistic regression model provided additional validation for these findings, demonstrating strong discriminatory performance with an area under the ROC curve of 85%. This result indicates a robust capacity to differentiate between ELISA-positive and ELISA-negative dogs, aligning with diagnostic models from other endemic regions, including Brazil, which reported comparable AUC values of approximately 84% [58]. Nevertheless, the false-negative rate was high (76.5%), suggesting that the model missed a substantial proportion of positive animals despite its overall good discriminative performance (AUC = 0.85). This underscores the persistent challenge of identifying the entire infected population, implying that a significant proportion of positive animals may remain undetected during routine screening [49,60].

This study demonstrates that combining rapid diagnostic tests with ELISA improves overall diagnostic performance by leveraging the complementary strengths of both assays. By using a multi-test approach, we captured a broader spectrum of infection status, particularly in asymptomatic dogs. This approach reduces the likelihood of misclassification and provides more reliable estimates of true infection prevalence in settings where no perfect reference test exists. Such integrated diagnostic strategies are increasingly recommended for surveillance of CanL in endemic regions [20]. Our findings align with recent research in Zambia, where the use of both rK39-based RDTs and ELISA provided strong evidence of true exposure, even when individual test results varied [16]. Such synergistic diagnostic protocols are increasingly recommended to improve the detection of subclinical infections that single-method screenings often miss [16,44].

The detection of competent phlebotomine vectors, particularly those from the genera *Phlebotomus* and *Sergentomyia*, together with measurable canine seroprevalence, provides additional evidence that *Leishmania* transmission is actively maintained in Baringo County [60]. Similar studies in Zambia confirmed the presence of sandflies supporting the likelihood of vector-borne transmission [16]. Although molecular confirmation of parasite species was beyond the scope of this study, these findings support the inclusion of canine surveillance within integrated One Health programs that combine reservoir monitoring, vector control, improved diagnostics, and strengthened human surveillance to reduce the risk of zoonotic visceral leishmaniasis.

### Study Limitations

This study has several limitations. First, the cross-sectional design precluded causal inference regarding the relationship between the identified risk factors and *Leishmania* seropositivity. It could not distinguish recently infected dogs in the pre-seroconversion phase from those with prior exposure. Second, diagnosis relied solely on serological assays without molecular or parasitological confirmation, preventing differentiation between active infection and past exposure. Furthermore, the lower sensitivity of the rapid diagnostic test (RDT) relative to ELISA suggests that RDT-based screening alone may underestimate the true burden of canine infection. The absence of molecular characterization also precluded identification of circulating *Leishmania* species, limiting interpretation of transmission dynamics and reservoir competence.

Third, although the sample size was adequate to identify the principal risk factors associated with canine *Leishmania* seropositivity, it limited the precision of estimates for some subgroups and the generalizability of the findings beyond the study area. Finally, reliance on owner-reported clinical information, purposive site selection, and the absence of integrated human, canine, and entomological data constrained comprehensive assessment of zoonotic transmission pathways and disease ecology. Future longitudinal One Health studies integrating molecular diagnostics, parasite characterization, vector surveillance, and concurrent human–animal sampling are needed to define transmission dynamics better and strengthen the evidence base for controlling canine and human leishmaniasis in Kenya and other endemic regions of East Africa.

## 5. Conclusions

This study demonstrates measurable exposure of domestic dogs to *Leishmania* parasites in Baringo County. It identifies breed, clinical status, housing conditions, and veterinary management as significant factors associated with *Leishmania* seropositivity. Although the cross-sectional serological design provides evidence of exposure rather than reservoir competence, the findings indicate active parasite circulation within the canine population and suggest that dogs may contribute to the epidemiology of visceral leishmaniasis in this endemic area of Baringo County. The absence of molecular species characterization and routine canine surveillance highlights important knowledge gaps that warrant further investigation. Therefore, strengthening integrated One Health surveillance through canine screening, improved serological and molecular diagnostics, vector control, and enhanced veterinary management will be essential to interrupt *Leishmania* transmission and reduce the burden of zoonotic visceral leishmaniasis in Kenya and other endemic regions of East Africa.

## Figures and Tables

**Table 1:** Frequency distribution of potential risk factors for *Leishmania* infection in dogs in Baringo County.

Variable	Categories	Frequency	%
Origin	Sabor	45	32.14
	Mbechot	40	28.57
	Rabai	27	19.29
	Chemolingot	16	11.43
	Kapkuikui	12	8.57
Age	Mature	58	41.43
	Old	47	33.57
	Young	35	25
Sex	Male	104	74.29
	Female	36	25.71
Housing	Roaming	82	58.57
	Within the compound	39	27.86
	Animal shed	11	7.86
	Kennel	8	5.71
Reason for keeping	Hunting	62	44.29
	Herding	45	32.14
	Security	26	18.57
	Pet	7	5
Breed	Local	123	87.86
	Mixed	17	12.14
Clinical status	None	105	75
	Emaciation	27	19.29
	Alopecia	5	3.57
	Diarrhea	3	2.14
Veterinary care	No	132	94.29
	Yes	8	5.71
Use of insecticide	No	133	95
	Yes	7	5
RDT Result	Negative	129	92.14
	Positive	11	7.86
Elisa result	Negative	123	87.86
	Positive	17	12.14

**Table 2:** Prevalence and factors associated with *Leishmania* seropositivity in dogs in Baringo County.

Variable	Categories	Positive	% Seropositivity	*p*-Value *
Sex	Female	3	17.6	0.560
	Male	14	82.4	
Age	Young	4	23.5	0.760
	Mature	6	**35.3**	
	Old	7	41.2	
Origin	Kapkuikui	4	23.5	**0.005**
	Mbechot	7	41.2	
	Rabai	5	29.4	
	Sabor	1	5.9	
	Chemolingot	0	0	
Veterinary care (Insecticide use)	No	16	94.1	1.00
	Yes	1	5.9	
Clinical status	Alopecia	2	11.8	**<0.001**
	Diarrhea	1	5.9	
	Emaciation	8	47.0	
	None	6	35.3	
Breed	Local	13	76.5	**0.010**
	Mixed	4	23.5	
Reason for keeping dog	Grazing	5	29.4	0.397
	Hunting	10	58.8	
	Pet	1	5.9	
	Security	1	5.9	
Housing	Kennel	1	5.9	**0.042**
	Roaming	15	88.2	
	Animal shed	0	0	
	Compound	1	5.9	

* ***p*****-values** were assessed using Fisher’s exact test; statistical significances are highlighted in bold.

**Table 3:** Multivariable logistic analysis of factors associated with *Leishmania* seropositivity in dogs.

Variable	Covariate	OR (95% CI)	*p*-Value
Clinical status	Yes	1.00	
	None	0.06 (0.01–0.29)	**<0.001**
Origin	Kapkuikui	1.00	
	Rabai	4.60 (0.78–27.15	0.092
Housing condition	Roaming dogs	1.00	
	Within the compound	0.013 (0.01–1.51)	0.103
Breed	Mixed	1.00	
	Local	11.91 (1.79–79.37)	**0.010**
Veterinary care	No	1.00	
	Yes	0.12 (0.01–1.69)	0.181

Statistical significances are highlighted in bold.

## Data Availability

The original contributions presented in this study are included in the article and its Supplementary Material. Further inquiries can be directed to the corresponding author(s).

## Supplementary Materials

The following supporting information can be downloaded at: https://www.mdpi.com/article/10.3390/vetsci13090857/s1.

## Abbreviations

The following abbreviations are used in this manuscript:

VL	Visceral Leishmaniasis
CL	Cutaneous Leishmaniasis
MCL	Mucocutaneous leishmaniasis
ZVL	Zoonotic visceral leishmaniasis
WHO	World Health Organization
PKDL	Post-kala-azar dermal leishmaniasis
NTD	Neglected Tropical Diseases
RDT	Rapid diagnostic Test
ELISA	Enzyme-linked immunosorbent assay
KEMRI	Kenya Medical Research Institute
KNBS	Kenya National Bureau of Statistics
