# Supplementary material for: Seroprevalence and Factors Associated with Canine Leishmaniasis in Baringo County, Kenya: Implications for an Integrated One Health Surveillance
Source: Vet Sci. 2026 Aug 24;13(9):857. doi: 10.3390/vetsci13090857 (PMC13611605; doi:10.3390/vetsci13090857)
Supplement: Supplementary file 1 [file vetsci-13-00857-s001.zip › Vetsci-4423184-Statistical analysis supplementarial.pdf]

STATISTICAL ANALYSIS FOR RESULTS

1- Descriptive statistics for factors associated with *Leishmania* seropositivity in dogs

- Dog age: Median: 3 years, IQR: [1.5 – 6 years]

```
. summarize dogage, detail

                           dogage
-------------------------------------------------------------
      Percentiles      Smallest
 1%            1              1
 5%            1              1
10%            1              1       Obs                 140
25%          1.5              1       Sum of wgt.         140

50%            3                      Mean           4.092857
                        Largest       Std. dev.      3.222887
75%            6             13
90%            9             14       Variance         10.387
95%           10             15       Skewness        1.35504
99%           15             15       Kurtosis       4.593604
```

- Dog sex

dogsex	Freq.	Percent	Cum.
female	36	25.71	25.71
male	104	74.29	100.00
Total	140	100.00	

- Housing dog

housingdog	Freq.	Percent	Cum.
Kennel	8	5.71	5.71
Roamingoutside	82	58.57	64.29
animalshed	11	7.86	72.14
withinthecompound	39	27.86	100.00
Total	140	100.00	

- Reason for dog

reasonfordo g	Freq.	Percent	Cum.
grazing	45	32.14	32.14
hunting	62	44.29	76.43
pet	7	5.00	81.43
security	26	18.57	100.00
Total	140	100.00	

- Breed

breed	Freq.	Percent	Cum.
local	123	87.86	87.86
mongrel	17	12.14	100.00
Total	140	100.00	

- Clinical status

clinicalstatus	Freq.	Percent	Cum.
alopecia	5	3.57	3.57
diarhhea	3	2.14	5.71
emaciation	27	19.29	25.00
none	105	75.00	100.00
Total	140	100.00	

- Vaccination

vaccination	Freq.	Percent	Cum.
no	132	94.29	94.29
yes	8	5.71	100.00
Total	140	100.00	

- Ectoparasiticide

ectoparasiticide	Freq.	Percent	Cum.
no	133	95.00	95.00
yes	7	5.00	100.00
Total	140	100.00	

- Origin

origin	Freq.	Percent	Cum.
Kapkuikui	12	8.57	8.57
Mbechot	40	28.57	37.14
Rabai	27	19.29	56.43
Sabor	45	32.14	88.57
chemolingot	16	11.43	100.00
Total	140	100.00	

- RDT result

RDTresult	Freq.	Percent	Cum.
+VE	11	7.86	7.86
-VE	129	92.14	100.00
Total	140	100.00	

- Elisa result

ELISAresult	Freq.	Percent	Cum.
NEGATIVE	123	87.86	87.86
POSITIVE	17	12.14	100.00
Total	140	100.00	

- Create dog age category [<=1: Young; >1 <5: Mature; >=5 old

dogage_cat	Freq.	Percent	Cum.
Young	35	25.00	25.00
Mature	58	41.43	66.43
Old	47	33.57	100.00
Total	140	100.00	

- RDT Prevalence Prev=7.79%; 95% IC [4.38 – 13.70]

```
. proportion rdt_positive

Proportion estimation                    Number of obs = 140
```

	Proportion	Std. err.	Logit [95% conf. interval]	
rdt_positive				
Negative	.9214286	.0227405	.8630526	.9561842
Positive	.0785714	.0227405	.0438158	.1369474

- ELISA prevalence Prev=12.14%, 95% IC [7.65 – 18.73

```
Proportion estimation                    Number of obs = 140
```

	Proportion	Std. err.	Logit [95% conf. interval]	
elisa_positive				
Negative	.8785714	.0276048	.8126571	.9234783
Positive	.1214286	.0276048	.0765217	.1873429

2- Bivariate analysis risk factors associated with *Leishmania* seroprevalence

- Elisa and dog age-cat: Fisher's exact= 0.760

	elisa_positive		
dogage_cat	Negative	Positive	Total
Young	31<br>88.57<br>25.20	4<br>11.43<br>23.53	35<br>100.00<br>25.00
Mature	52<br>89.66<br>42.28	6<br>10.34<br>35.29	58<br>100.00<br>41.43
Old	40<br>85.11<br>32.52	7<br>14.89<br>41.18	47<br>100.00<br>33.57
Total	123<br>87.86<br>100.00	17<br>12.14<br>100.00	140<br>100.00<br>100.00

- Elisa result and vaccination Fisher's exact = 1.000

vaccination	elisa_positive Negative	Positive	Total
no	116<br>87.88<br>94.31	16<br>12.12<br>94.12	132<br>100.00<br>94.29
yes	7<br>87.50<br>5.69	1<br>12.50<br>5.88	8<br>100.00<br>5.71
Total	123<br>87.86<br>100.00	17<br>12.14<br>100.00	140<br>100.00<br>100.00

- Elisa and ectoparasiticide; Fisher's exact = 1.000

ectoparasiticide	elisa_positive Negative	Positive	Total
no	117<br>87.97<br>95.12	16<br>12.03<br>94.12	133<br>100.00<br>95.00
yes	6<br>85.71<br>4.88	1<br>14.29<br>5.88	7<br>100.00<br>5.00
Total	123<br>87.86<br>100.00	17<br>12.14<br>100.00	140<br>100.00<br>100.00

- Elisa and clinical status; Fisher's exact <0.001

clinicalstatus	elisa_positive Negative	Positive	Total
alopecia	3<br>60.00<br>2.44	2<br>40.00<br>11.76	5<br>100.00<br>3.57
diarhhea	2<br>66.67<br>1.63	1<br>33.33<br>5.88	3<br>100.00<br>2.14
emaciation	19<br>70.37<br>15.45	8<br>29.63<br>47.06	27<br>100.00<br>19.29
none	99<br>94.29<br>80.49	6<br>5.71<br>35.29	105<br>100.00<br>75.00
Total	123<br>87.86<br>100.00	17<br>12.14<br>100.00	140<br>100.00<br>100.00

- Elisa result and breed; Fisher's exact = 0.130

breed	elisa_positive Negative	Positive	Total
local	110	13	123
	89.43	10.57	100.00
	89.43	76.47	87.86
mongrel	13	4	17
	76.47	23.53	100.00
	10.57	23.53	12.14
Total	123	17	140
	87.86	12.14	100.00
	100.00	100.00	100.00

- Reason for dog; Fisher's exact = 0.397

reasonford og	elisa_positive Negative	Positive	Total
grazing	40	5	45
	88.89	11.11	100.00
	32.52	29.41	32.14
hunting	52	10	62
	83.87	16.13	100.00
	42.28	58.82	44.29
pet	6	1	7
	85.71	14.29	100.00
	4.88	5.88	5.00
security	25	1	26
	96.15	3.85	100.00
	20.33	5.88	18.57
Total	123	17	140
	87.86	12.14	100.00
	100.00	100.00	100.00

- Housing dog; Fischer's exact = 0.042

housingdog	elisa_positive Negative	Positive	Total
Kennel	7	1	8
	87.50	12.50	100.00
	5.69	5.88	5.71
Roamingoutside	67	15	82
	81.71	18.29	100.00
	54.47	88.24	58.57
animalshed	11	0	11
	100.00	0.00	100.00
	8.94	0.00	7.86
withinthecompound	38	1	39
	97.44	2.56	100.00
	30.89	5.88	27.86
Total	123	17	140
	87.86	12.14	100.00
	100.00	100.00	100.00

- Elisa result and dog sex; Fisher's exact = 0.560

dogsex	elisa_positive Negative	Positive	Total
female	33	3	36
	91.67	8.33	100.00
	26.83	17.65	25.71
male	90	14	104
	86.54	13.46	100.00
	73.17	82.35	74.29
Total	123	17	140
	87.86	12.14	100.00
	100.00	100.00	100.00

housingdog	elisa_positive Negative	Positive	Total
Kennel	7	1	8
	87.50	12.50	100.00
	5.69	5.88	5.71
Roamingoutside	67	15	82
	81.71	18.29	100.00
	54.47	88.24	58.57
animalshed	11	0	11
	100.00	0.00	100.00
	8.94	0.00	7.86
withinthecompound	38	1	39
	97.44	2.56	100.00
	30.89	5.88	27.86
Total	123	17	140
	87.86	12.14	100.00
	100.00	100.00	100.00

3- Stepwise logistic regression analysis

```
Logistic regression                                     Number of obs =    113
                                                        LR chi2(5)    =  28.12
                                                        Prob > chi2   = 0.0000
Log likelihood = -33.793316                             Pseudo R2     = 0.2938
```

elisa_positive	Odds ratio	Std. err.	z	P>|z|	[95% conf. interval]	
clinicalstatus_n						
none	.063069	.0489714	-3.56	0.000	.0137683	.2889028
origin_n						
Rabai	4.603624	4.16808	1.69	0.092	.7806016	27.15002
housingdog_n						
withinthecompound	.1314004	.1637385	-1.63	0.103	.0114265	1.511059
breed_n						
mongrel	11.91665	11.5286	2.56	0.010	1.789246	79.36662
vaccination_n						
yes	.1185246	.1889067	-1.34	0.181	.0052137	2.694448
_cons	.6091686	.2770368	-1.09	0.276	.2498213	1.485408

Overall model performance

- **Number of observations:** 113
- **Likelihood Ratio (LR) chi²(5) = 28.12**
- **$p < 0.001$**

This indicates that the model as a whole is statistically significant, meaning that the included predictors jointly explain ELISA positivity.

- **Pseudo $R^2 = 0.2938$**

Approximately 29.4% of the variability in ELISA positivity is explained by the variables included in the model.

Interpretation of each variable

1. Clinical status

- **OR = 0.063**
- **95% CI: 0.014 – 0.289**
- **$p < 0.001$**

Dogs without clinical signs had significantly lower odds of ELISA positivity compared with dog with alopecia. This association is statistically significant.

2. Origin

Category:

Rabai

- **OR = 4.60**
- **95% CI: 0.78 – 27.15**
- **p = 0.092**

Dogs originating from Rabai had approximately 4.6 times higher odds of ELISA positivity compared with the dogs origination from Kapkuikui

However:

- the confidence interval is wide,
- it includes 1,
- and p > 0.05.

Therefore, this association is **not statistically significant**, although it suggests a possible trend.

3. Housing of dog

Category:

Within the compound

- **OR = 0.13**
- **95% CI: 0.011 – 1.51**
- **p = 0.103**

Dogs housed within the compound had lower odds of ELISA positivity compared with the reference housing Kennel.

However, the result is not statistically significant.

4. Breed

Category:

mongrel

- **OR = 11.92**
- **95% CI: 1.79 – 79.37**
- **p = 0.010**

Mongrel dogs had nearly 12 times higher odds of ELISA positivity compared with the local's.

This association is statistically significant.

However, the confidence interval is very wide, suggesting:

- limited precision,
- likely due to small sample size or sparse data.

5. Vaccination status

Category:

yes

- **OR = 0.12**
- **95% CI: 0.005 – 2.69**
- **p = 0.181**

Vaccinated dogs appeared to have lower odds of ELISA positivity.

However, this association was not statistically significant.

Writing

Multivariable logistic regression identified breed and clinical status as significant predictors of ELISA positivity among dogs. Mongrel dogs had significantly higher odds of seropositivity compared with the local's (OR = 11.92; 95% CI: 1.79–79.37; p = 0.010). Conversely, dogs without clinical signs had significantly lower odds of ELISA positivity (OR = 0.06; 95% CI: 0.01–0.29; p < 0.001). Although dogs from Rabai and those housed within compounds showed trends toward increased and reduced odds respectively, these associations did not reach statistical significance.

Goodnest of fit of the model:

```
Goodness-of-fit test after logistic model
Variable: elisa_positive

      Number of observations =    113
Number of covariate patterns =     10
              Pearson chi2(4) =   5.77
                  Prob > chi2 = 0.2170
```

The goodness-of-fit test indicated that the logistic regression model adequately fit the data (Pearson χ^2 = 5.77, df = 4, p = 0.217).

ROC curve if the model:

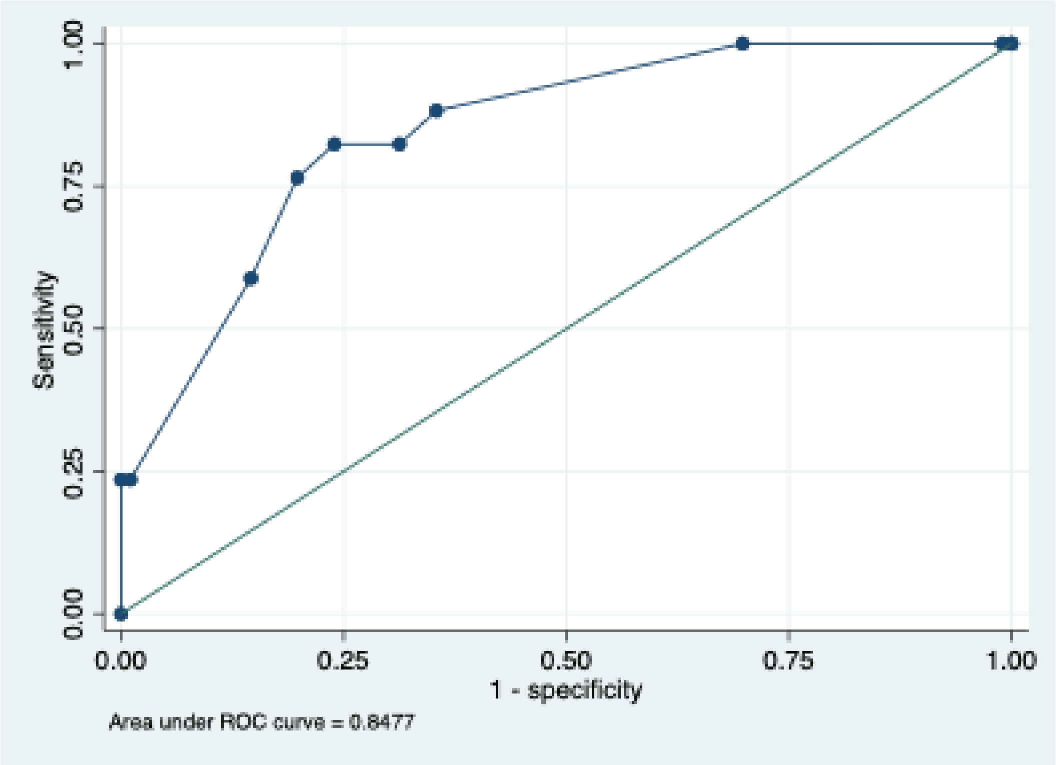

The logistic regression model demonstrated excellent discriminatory performance, with an area under the ROC curve (AUC) of 0.85, indicating good ability to distinguish ELISA-positive from ELISA-negative dogs.

Classification table of the model

Logistic model for elisa_positive

Classified	True D	True ~D	Total
+	4	0	4
-	13	96	109
Total	17	96	113

Classified + if predicted Pr(D) >= .5
True D defined as elisa_positive != 0

Sensitivity	Pr(+| D)	23.53%
Specificity	Pr(-|~D)	100.00%
Positive predictive value	Pr(D| +)	100.00%
Negative predictive value	Pr(~D| -)	88.07%
False + rate for true ~D	Pr(+|~D)	0.00%
False - rate for true D	Pr(-| D)	76.47%
False + rate for classified +	Pr(~D| +)	0.00%
False - rate for classified -	Pr(D| -)	11.93%
Correctly classified		88.50%

Confusion Matrix Interpretation

Predicted	True Positive (D)	True Negative (~D)	Total
Positive (+)	4	0	4
Negative (-)	13	96	109
Total	17	96	113

Where:

- **D** = ELISA positive
- **~D** = ELISA negative

Key Performance Indicators

1. Sensitivity = 23.53%

$\text{Sensitivity} = \frac{4}{17}$

This means the model correctly identified only:

- **23.5% of ELISA-positive dogs**.

Interpretation

The model has **low sensitivity**, meaning it misses many positive cases.

Indeed:

- 13 out of 17 positive dogs were incorrectly classified as negative.

---

2. Specificity = 100%

$\text{Specificity} = \frac{96}{96}$

The model correctly identified:

- **all ELISA-negative dogs**.

Interpretation

The model is extremely good at ruling out negatives.

There were:

- **no false positives**.

3. Positive Predictive Value (PPV) = 100%

Every dog predicted as positive was truly positive.

4. Negative Predictive Value (NPV) = 88.07%

Among dogs predicted negative:

- 88.1% were truly negative.

1. **Error Rates**

False Negative Rate = 76.47%

This is high.

The model fails to detect many truly infected dogs.

False Positive Rate = 0%

No negative dogs were incorrectly classified as positive.

2. **Overall Accuracy**

Correctly classified = 88.50%

The model correctly classified:

- 100 out of 113 dogs.

3. **Important caution**

Although:

- overall accuracy is high (88.5%),
- and specificity is excellent,

the model has **poor sensitivity**.

This likely occurs because:

- ELISA-positive dogs are relatively few (17 positives),
- creating class imbalance.

Thus, the model tends to predict most dogs as negative.

4. **Scientific interpretation**

Your model is:

- very conservative,
- highly specific,
- but not sensitive enough for screening purposes.

In epidemiological or surveillance contexts, missing positive animals may be problematic.

The logistic regression model correctly classified 88.5% of observations. The model demonstrated excellent specificity (100%) and positive predictive value (100%), but low sensitivity (23.5%), indicating limited ability to identify ELISA-positive dogs. The false negative rate was high (76.5%), suggesting that a substantial proportion of positive animals were missed by the model despite its overall good discriminatory performance (AUC = 0.85).

Agreement between RDT and Elisa (Kappa stat)

```
. kap rdt_positive elisa_positive
```

Agreement	Expected agreement	Kappa	Std. err.	Z	Prob>Z
95.71%	81.91%	0.7631	0.0821	9.29	0.0000

The RDT results are strongly consistent with ELISA results.

This suggests that the rapid diagnostic test may be a reliable alternative or screening tool compared with ELISA in this dog population.

However:
Kappa is not perfect (not close to 1), meaning some discordant results still exist.

Agreement between the rapid diagnostic test (RDT) and ELISA was substantial, with a Cohen's Kappa coefficient of 0.763 ($p < 0.001$). The overall observed agreement between the two diagnostic methods was 95.7%, indicating strong concordance beyond chance.

Cross tabulation

rdt_positive	elisa_positive Negative	Positive	Total
Negative	123	6	129
	95.35	4.65	100.00
	100.00	35.29	92.14
Positive	0	11	11
	0.00	100.00	100.00
	0.00	64.71	7.86
Total	123	17	140
	87.86	12.14	100.00
	100.00	100.00	100.00

rdt_positive (Rapid Diagnostic Test result)
elisa_positive (ELISA result)

- Concordant results
    - True negatives

123 dogs were negative by both RDT and ELISA.

True positives

11 dogs were positive by both methods.

This explains the very high observed agreement previously seen in the Kappa analysis.

- Discordant results

False negatives

6 dogs were: negative by RDT, but positive by ELISA. This indicates that the RDT missed some infected/seropositive dogs.

False positives

No dogs were RDT positive and ELISA negative.

Thus: the RDT produced no false positives in this dataset.

Diagnostic performance of the RDT

(Using ELISA as reference standard)

Sensitivity
= 64.7\%

The RDT correctly identified: approximately 65% of ELISA-positive dogs.

Specificity = 100\%

The RDT perfectly identified ELISA-negative dogs.

Positive Predictive Value (PPV)

PPV = 100%

All RDT-positive dogs were confirmed positive by ELISA.

Negative Predictive Value (NPV)

NPV= 95.3\%

Among RDT-negative dogs: 95.3% were truly ELISA negative.

Epidemiological interpretation

The RDT demonstrated: excellent specificity, excellent PPV, good overall agreement with ELISA, but moderate sensitivity.

This means: a positive RDT result is highly reliable, but a negative RDT result does not completely exclude infection.

Therefore, some infected dogs may be missed if only RDT is used.

Compared with ELISA, the rapid diagnostic test (RDT) demonstrated a sensitivity of 64.7% and a specificity of 100%. All RDT-positive samples were confirmed positive by ELISA, yielding a positive predictive value of 100%. However, six ELISA-positive dogs tested negative by RDT, indicating that the RDT failed to detect a proportion of seropositive animals.

rdt_positi	elisa_positive		
ve	Negative	Positive	Total
Negative	123	6	129
	95.35	4.65	100.00
	100.00	35.29	92.14
Positive	0	11	11
	0.00	100.00	100.00
	0.00	64.71	7.86
Total	123	17	140
	87.86	12.14	100.00
	100.00	100.00	100.00
